# Effects of N^6^‑Methyladenosine (m^6^A) and 5‑Methylcytosine (m^5^C) Modifications in the Guide Region of CRISPR RNA on Cas12a Nuclease Activity

**DOI:** 10.1021/acs.bioconjchem.5c00498

**Published:** 2025-12-05

**Authors:** Bhoomika Pandit, Emmett Hanson, Hilal Dagci, Qingying Yang, Mehmet V. Yigit, Maksim Royzen

**Affiliations:** † Department of Chemistry, 1084 University at Albany, State University of New York, 1400 Washington Avenue, Albany, New York 12222, United States; ‡ The RNA Institute, University at Albany, State University of New York, 1400 Washington Avenue, Albany, New York 12222, United States

## Abstract

CRISPR–Cas12a is a versatile biosensing platform that detects sequence-specific DNA or RNA targets via a CRISPR RNA (crRNA) guide. While Cas12a’s specificity is dictated by its crRNA, chemical modifications within the crRNA can influence nuclease performance. Here, we examined the effects of two well-known RNA modifications, N^6^-methyladenosine (m^6^A) and 5-methylcytosine (m^5^C), introduced into the different positions of the guide region of a crRNA. Melting temperature (*T*_m_) analysis showed that m^6^A had a minimal impact on RNA–DNA duplex stability. In contrast, the incorporation of m^5^C residues stabilized the duplex. Using a fluorescence recovery assay, we found that both modifications preserved Cas12a’s nuclease activity, indicating that small thermodynamic shifts in duplex formation are insufficient to disrupt its catalytic function. Despite the greater *T*_m_ increase with m^5^C, m^6^A incorporation led to a faster fluorescence recovery rate than that with m^5^C.

## Graphical abstract

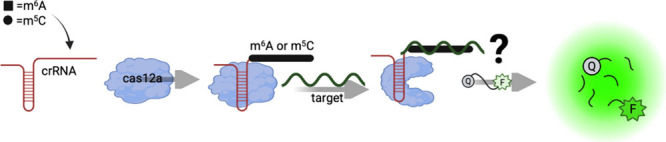

## Introduction

CRISPR-Cas12a has emerged as a powerful tool for biosensing applications.^1,2^ It offers a flexible platform by detecting sequence-specific RNA and DNAs through its CRISPR RNA (crRNA) element.^3,4^ Upon recognition of a target complementary to its crRNA, Cas12a becomes activated and exhibits the indiscriminate cleavage of single-stranded DNA through its nuclease property. This property has been harnessed in diagnostic platforms such as SHERLOCK and DETECTR, which employ short single-stranded DNA molecules labeled with fluorescent and quencher (F-Q) pairs to signal target detection.^5–9^ The F-Q ssDNA approach has proven to be a reliable and sensitive method for demonstrating Cas12a’s target recognition and activity. Furthermore, the system’s specificity can be readily programmed by simply replacing the crRNA within the Cas12a-crRNA complex, making Cas12a-based diagnostics highly adaptable for diverse biological sensing needs.^10,11^ Significant efforts have been devoted to understanding the role of crRNA in modulating Cas12a’s nuclease activity. In particular, chemical modifications to the crRNA can influence the overall activity of Cas12a, a property we investigated in the present study.^12,13^

Here, we have studied two well-known RNA modifications, *N* ^6^-methyladenosine (m^6^A) and 5-methylcytosine (m^5^C), on Cas12a’s nuclease activity, shown in Scheme 1. The former is the most abundant internal modification of mRNAs and noncoding RNAs.^14^ It is typically found in 5′-UTR and near the start codon. m^6^A is known to regulate translation of mRNA by recruiting m^6^A reader proteins, such as YTHDF1, YTHDF2, and YTHDF3, as well as YTHDC1 and YTHDC2, which promote mRNA translation.^15^ Additional regulation stems from m^6^A’s ability to repel certain RNA-binding proteins, forming a secondary layer of regulation of mRNA translation.^16^ On the other hand, m^5^C is a post-transcriptional RNA modification that is most enriched in tRNAs and rRNAs.^17^ It is known to serve very different biological functions. For example, m^5^C modification contributes to tRNA stability.^18^ It is also important for rRNA assembly.^19^ Dysregulation of proper m^5^C modifications is linked to pathological processes, including nervous system disorders and cancers.^20,21^

**Scheme 1:**
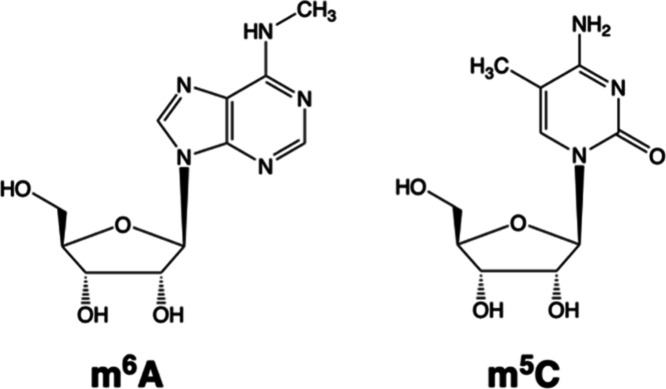
Chemical Structures of m^6^A and m^5^C RNA Modifications

Very little is known about the impacts of m^6^A and m^5^C on CRISPR-Cas12a nuclease activity. Unlike other well-known RNA modifications, such as *N* ^*1*^-methyladenosine (m^1^A) and 3-methylcytosine (m^3^C), m^6^A and m^5^C do not directly perturb the Watson–Crick base pairing of the DNA-RNA duplex, the formation of which is crucial when crRNA recognizes its target. Instead, m^6^A and m^5^C have more subtle either stabilizing or destabilizing effects on the DNA-RNA duplex stability. Previous studies by Kool et al. have shown that m^6^A pairing in the double-stranded context is accompanied by the methylamino group rotating from its energetically preferred *syn* geometry on the Watson–Crick face to the higher-energy anti conformation.^22^ This results in the positioning of the methyl group in the major groove of the duplex. Thermodynamic measurements have determined that a single m^6^A residue causes duplex destabilization by 0.5–1.7 kcal/mol.

The impact of the m^5^C modification on the duplex structure has been investigated by a number of research groups. Although m^5^C does not hinder canonical Watson–Crick base-pairing interactions, this modification can, in fact, cause significant conformational changes in certain RNA and DNA sequence contexts. These changes typically lead to an increased duplex stability. Mechanistically, it is thought to be due to the ability of m^5^C to induce local distortions of the phosphate backbone and a C3′-*endo* to C2′-*endo* sugar pucker.^23–25^

The impact of RNA modifications on CRISPR–Cas12a activity has been widely examined.^26–28^ In particular, it was recently found that site-specific modification of guide RNA with m^6^A or m^1^A in the stem-loop region can dramatically impact Cas12a binding. This finding was utilized to regulate the nuclease activity. Previously, we investigated this in a CRISPR-Cas9 system.^28^ We found that RNA modifications that are known to stabilize the DNA-RNA duplex decreased the nuclease activity, while m^6^A improved the on-target activity. This was counterintuitive as m^6^A is known to destabilize the DNA-RNA duplex. Intrigued by this finding, we investigated how m^6^A modification in the guide region influences the CRISPR–Cas12a nuclease activity. We also examined m^5^C modification, known to stabilize the RNA–DNA duplex, to determine whether it affects Cas12a nuclease activity.

## Results and Discussion

To assess the impacts of m^6^A and m^5^C modifications, we synthesized five CRISPR RNA strands targeting the GFP gene, Table 1. **crRNA1** (wild type) is a guide RNA that does not contain any modifications. **crRNA2** contains two m^6^A modifications in the PAM-distal guide region (italicized letters with star). **crRNA3–5** contain three m^5^C modifications at different positions (PAM-distal, PAM-medial, and PAM-proximal, italicized letters with star) of the guide region, Table 1. The RNAs were prepared by solid-phase synthesis and purified by preparative PAGE. Purity was assessed by analytical gel electrophoresis and ESI-MS, Figures S4–S14.

**Table 1:** CrRNA Sequences Synthesized and Used^a^

**crRNA1**	5′AAUUUCUACUCUUGUAGAUCGUCGCCGUCCAGCUCGACC3′
**crRNA2**	5′AAUUUCUACUCUUGUAGAUCGUCGCCGUCCA*GCUCGA*CC3′
**crRNA3**	5′AAUUUCUACUCUUGUAGAU**CGUCGCCGUCC**AGC*UC*GAC*C3′
**crRNA4**	5′AAUUUCUACUCUUGUAGAUCGUCGCC*GUC*C*AGCUCGACC3′
**crRNA5**	5′AAUUUCUACUCUUGUAGAU C*GUC*GC*CGUCCAGCUCGACC3′

a m^6^A and m^5^C modifications are represented italicized letters with a star.

Prior to assessing the effects of these RNAs on Cas12a nuclease activity, we examined how modifications in each guide region influence the RNA-DNA duplex stability. In addition to the full crRNAs, we synthesized 20-nt RNAs corresponding solely to the guide region, bold regions in the crRNA sequences provided in Tables 1 and S1, which, in the CRISPR-Cas12a system, are responsible for recognizing the target DNA. Our guide sequence contains only two adenosines. Therefore, we synthesized **20-nt-crRNA1**, containing canonical nucleotides and **20-nt-crRNA2** where both adenosines were replaced with m^6^A. The guide sequence contains ten cytidines. So, we prepared **20-nt-crRNA3**, **20-nt-crRNA4**, and **20-nt-crRNA5** each containing three m^5^C modifications placed at different positions, Table 1. We performed the UV-melting *T*_m_ studies using a complementary DNA (target sequence) to investigate the impact of m^6^A and m^5^C modifications on the stability of the RNA-DNA duplex. The normalized *T*_m_ curves are shown in Figure S1, and the detailed melting temperature data are summarized in Table 2. From these data, we observed a minimal impact of m^6^A on duplex stability. In general, m^6^A would be expected to cause small destabilization to a DNA-RNA duplex.^22^ However, the impact is also thought to be highly sequence-dependent.^29^ Meanwhile, the incorporation of three m^5^C residues stabilized the RNA-DNA duplex, which resulted in higher *T*_m_ values. This is also consistent with the previously reported studies involving m^5^C.^24,25^

**Table 2:** Types and Numbers of Modifications, Their Locations, and Calculated and Measured Molecular Weights Are Provided^a^

RNAs	Modifications	Location	Calculated MW (Da)	Observed MW (Da)	*T*_m_ (°C)	Δ*T*_m_ (°C)
**crRNAI**	wildtype		12294.6	12294.6	81.6	
**crRNA2**	Two m^6^A	PAM-distal	12328.5	12328.5	82.1	+ 0.5
**crRNA3**	Three m^5^C	PAM-distal	12342.4	12343.4	85.8	+ 4.2
**crRNA4**	Three m^5^C	PAM-medial	12342.4	12343.4	85.5	+ 3.9
**crRNAS**	Three m^5^C	PAM-proximal	12342.4	12343.3	84.5	+ 2.9

a ChemDraw software was used to determine the calculated molecular weights, whereas ESI-MS was used to determine the observed molecular weights.

After measuring the *T*_m_ values of the guide region with the target DNA, we assessed how the full crRNA constructs influence Cas12a-mediated nuclease activity. Each crRNA was first complexed with the Cas12a enzyme, and a complementary DNA target was introduced to activate the complex. Upon activation, the Cas12a–crRNA–target assembly is expected to indiscriminately cleave the F-Q probe introduced into the reaction. This cleavage separates the fluorophore (F) from the quencher (Q), thereby disrupting FRET and restoring fluorescence, Scheme 2.

**Scheme 2:**
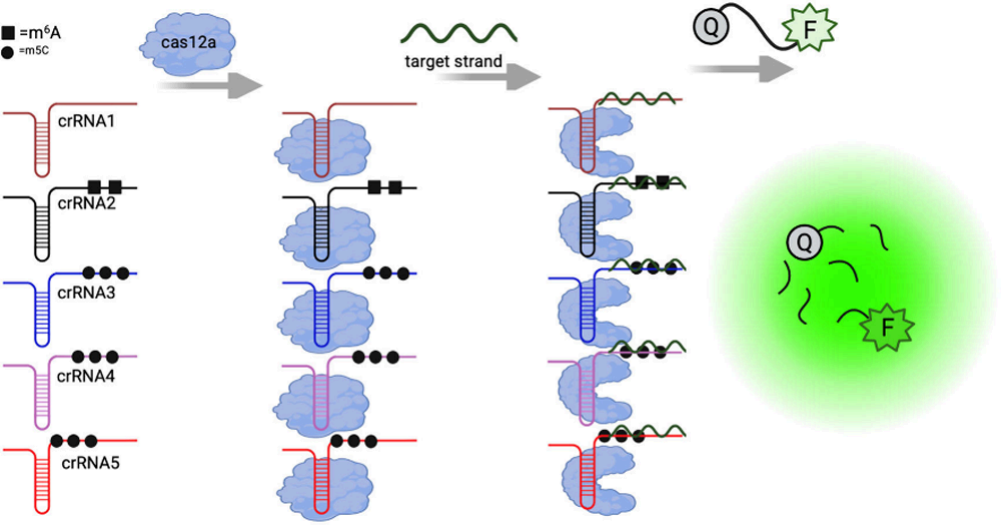
Illustration of Cas12a Activation Using Native crRNA (crRNA1), m^6^A-Modified crRNA (crRNA2, black squares), and m^5^C-Modified crRNAs (crRNA3–crRNA5, circles)^a^

a The number of modifications corresponds to the number of shapes illustrated on each crRNA. Upon activation, Cas12a degrades the F–Q probe, leading to fluorescence recovery. DNA target sequence is 5′-GGTCG­AGCTGGACG­GCGACG-3′. Fluorophore–quencher pair: FAM (6-carboxyfluorescein; excitation/emission: 495/520 nm) and BHQ1 (Black Hole Quencher 1; quenching range: 480–580 nm) were used in the F–Q probe (5′-FAM-TTTTT­TTTTT-BHQ1-3′).

Fluorescence measurements were performed with Cas12a complexed with each crRNA and activated by the target DNA. The fluorescence spectra (Figure S2) showed that all five crRNAs efficiently activated Cas12a and cleaved the F–Q probes. In the absence of the target, fluorescence remained quenched, whereas, in the presence of the target, fluorescence increased dramatically 40 min after addition of the F–Q probe.

To compare the fluorescence recoveries for all five crRNA constructs, we plotted the recoveries at multiple time points. Fluorescence kinetics at 520 nm for each Cas12a–crRNA complex (Figure S2), along with the end-point bar graphs in Figure 1, shows that all modified crRNAs exhibited the same recovery trend as the wild-type RNA, with no statistically significant differences under these conditions.

**Figure 1:**
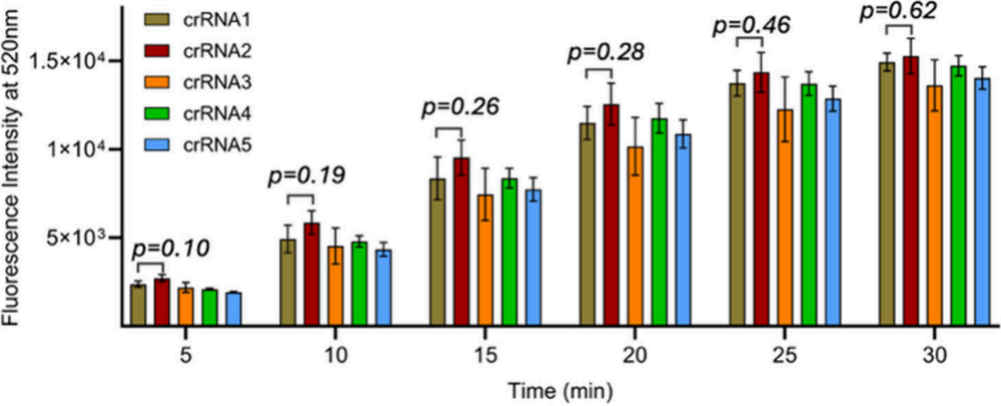
Fluorescence recovery intensity at 520 nm was measured at 5, 10, 15, 20, 25, and 30 min after the addition of the target. The experiments were performed in triplicate, and data are presented as mean ± SD. Statistical significance was evaluated using a student’s *t*-test. A *p*-value >0.1 indicates no statistically significant difference.

Although the results under these conditions suggest that neither m^6^A nor m^5^C modifications had major enhancing or disruptive effects on Cas12a activity, we further examined the initial recovery rates of each construct. To do this, we measured the fluorescence recoveries of each RNA construct side-by-side and compared the initial reaction rates within the first seven minutes.

Figure 2 and Table 3 illustrate that crRNA2, containing m^6^A, exhibited a higher fluorescence recovery rate compared to those of crRNA3, crRNA4, and crRNA5, which contain m^5^C modifications. The differences are subtle, and the stabilization of the DNA–RNA duplex by m^5^C through *T*_m_ measurements is not proportional to the recovery rate. In contrast, m^6^A showed the smallest increase in *T*_m_ but resulted in an initial recovery rate faster than those of the other tested constructs. This is analogous with our previous observation with the CRISPR-Cas9 system, where m^6^A also showed the most efficient activity.^28^

**Figure 2:**
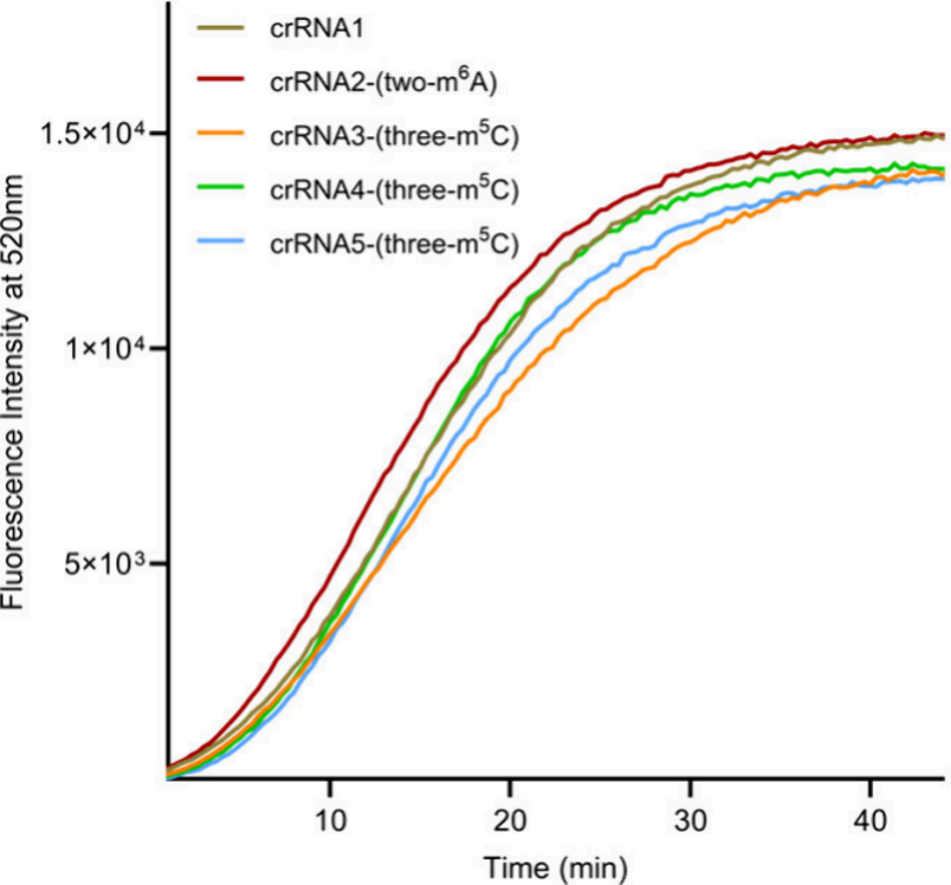
Fluorescence kinetics of Cas12a’ nuclease activity with crRNAs (2–4) containing m^6^A and m^5^C modifications and crRNA1 with no modifications. The experiments are performed in triplicate.

**Table 3:** Initial Reaction Rated Observed with All Five crRNAs, Showing Faster Kinetics with m^6^A Modification

Initial reaction rate between minutes 0–7 (unit = RFU/sec)				
crRNA1	crRNA2	crRNA3	crRNA4	crRNA5
2.99	3.50	2.39	2.05	1.61

Despite the moderate increase in melting temperature observed for m^5^C-containing crRNAs, we detected no substantial impact on Cas12a’s nuclease activity. This finding suggests that duplex stability alone does not govern target recognition or catalysis. Instead, Cas12a activity likely depends on precise protein–RNA and protein–DNA interactions along with the conformational transitions occurring during R loop formation.

To determine whether the fluorescence recovery observed with all five crRNAs is due to the formation of the Cas12a–crRNA–target complex, we conducted a fluorescence-quenching (F-Q) study under four conditions: (i) Cas12a with crRNA only (Cas12a–crRNA complex), (ii) Cas12a with target only (no crRNA), (iii) crRNA with target only (no Cas12a), and (iv) Cas12a with crRNA and target (Cas12a–crRNA–target complex), as shown in Figures 3 and S3.

**Figure 3:**
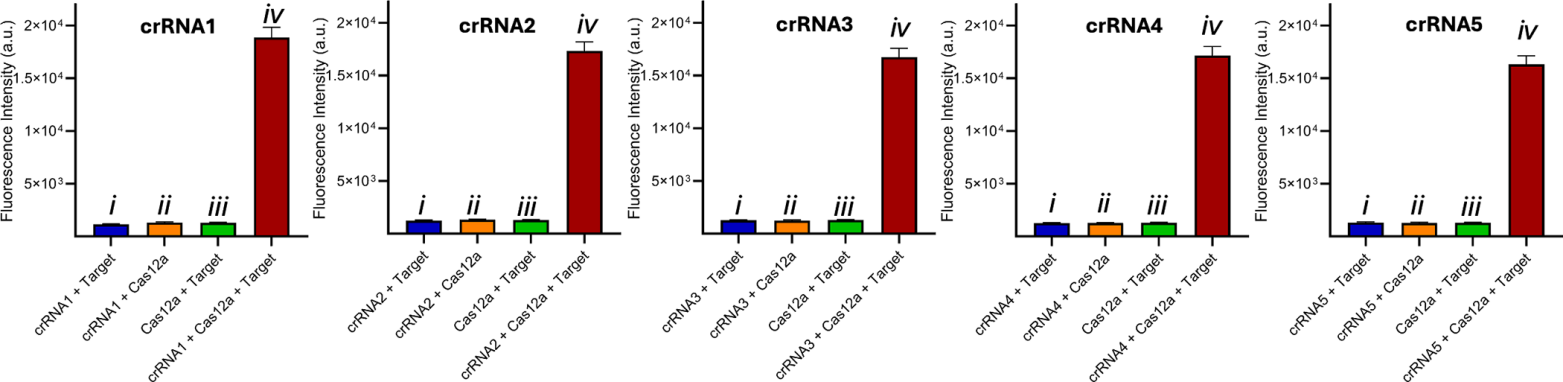
Cas12a nuclease activity with all crRNAs (crRNA1–crRNA5) containing m^6^A and m^5^C modifications, showing a fluorescence recovery intensity at 520 nm at the 40 min time point. Experiments were performed under four conditions: (i) Cas12a with crRNA only (Cas12a–crRNA complex), (ii) Cas12a with target only (no crRNA), (iii) crRNA with target only (no Cas12a), and (iv) Cas12a, crRNA, and target (Cas12a–crRNA–target complex). Fluorescence recovery occurs only when all three components are present. The experiments were performed in triplicate, and data are presented as mean ± SD.

We found that fluorescence recovery and F-Q cleavage occurred only when all three components (Cas12a, crRNA, and target) were present, suggesting formation of Cas12a-crRNA-target complex with all five crRNAs (Table 4).

**Table 4:** Cas12a Cleavage Activity Is Observed Only When All Three Components Are Present^a^

crRNA(1–5)	*i*	*ii*	*iii*	*iv*
crRNA	+	+	-	+
cas12a	-	+	+	+
target	+	-	+	+
**Result**	-	-	-	+

a Experiments were performed under four conditions: (i) Cas12a with crRNA only (Cas12a–crRNA complex), (ii) Cas12a with target only (no crRNA), (iii) crRNA with target only (no Cas12a), and (iv) Cas12a, crRNA, and target (Cas12a–crRNA–target complex).

## Conclusions

In conclusion, we synthesized five crRNAs: one native, one modified with m^6^A, and three modified with m^5^C. Melting temperature analysis indicated that the three m^5^C modifications increased RNA–DNA duplex stability, whereas the two m^5^A modifications had a minimal impact. To determine whether this enhanced stability affected Cas12a activity, Cas12a–crRNA complexes were prepared with all five crRNAs, and their nuclease activities were compared. Our results showed that, despite differences in duplex stability, there was no significant change in recovered fluorescence intensities. The initial rate of fluorescence recovery, however, was slower for crRNAs with m^5^C modifications, while crRNA containing m^6^A exhibited a modest increase in the initial recovery rate. Although the m^5^C-modified crRNAs exhibited moderately increased melting temperatures relative to the unmodified control, the Cas12a nuclease activity remained largely unaffected. This observation suggests that alterations in duplex thermodynamics alone are insufficient to perturb the catalytic function of Cas12a. The enzyme’s activity likely depends more critically on specific protein–RNA and protein–DNA interactions, as well as on conformational transitions during loop formation, rather than on small changes in overall duplex stability.

## Supplementary Material



## Data Availability

The detailed experimental procedure and additional data are available in Supporting Information.

## Notes

The detailed experimental procedure, list of materials, oligonucleotide sequences, and additional data (PDF)
