# Supplementary material for: Effects of N6‑Methyladenosine (m6A) and 5‑Methylcytosine (m5C) Modifications in the Guide Region of CRISPR RNA on Cas12a Nuclease Activity
Source: Bioconjug Chem. 2025 Dec 5;36(12):2551–6. doi: 10.1021/acs.bioconjchem.5c00498 (PMC12715751; doi:10.1021/acs.bioconjchem.5c00498)
Supplement: Supplementary file 1 [file bc5c00498_si_001.pdf]

SUPPORTING INFORMATION

Effects of N^6-Methyladenosine (m^{6}A) and 5-Methylcytosine (m^{5}C) Modifications in the Guide Region of CRISPR RNA on Cas12a Nuclease Activity

Bhoomika Pandit [†], Emmett Hanson[†], Hilal Dagci[†], Qingying Yang[†], Mehmet V. Yigit[*,†, ‡] and Maksim Royzen[*,†, ‡]

† Department of Chemistry,
University at Albany, State University of New York,
1400 Washington Avenue, Albany, New York 12222, United States.

‡ The RNA Institute,
University at Albany, State University of New York,
1400 Washington Avenue, Albany, New York 12222, United States.

*Correspondence:
Tel: (1) 518-442-3002
myigit@albany.edu
and
mroyzen@albany.edu

Keywords: fluorescence • CRISPR-Cas12a • N^6-Methyladenosine • 5-Methylcytosine • RNA• DNA

Materials and Methods.

All oligonucleotide solid phase syntheses were done on a 1.0 μmol scale using the Oligo-800 synthesizer (Azco Biotech, Oceanside, CA, USA). Solid phase syntheses were performed on control-pore glass (CPG-1000) purchased from Glen Research (Sterling, VA, USA). The phosphoramidites of m^5C and m^6A were also purchased from Glen Research (Sterling, VA, USA). Other oligonucleotide solid phase synthesis reagents were obtained from ChemGenes Corporation (Wilmington, MA, USA). Phosphoramidites (TBDMS as the 2′-OH protecting group): rA was N-Bz protected, rC was N-Ac protected and rG was N-iBu protected. A, C, G, U phosphoramidites were dissolved in anhydrous acetonitrile (0.07 M) directly before use. m^6A and m^5C phosphoramidites were dissolved in anhydrous acetonitrile (0.15 M) directly before use. Coupling step was done using 5-ethylthio-1H-tetrazole solution (0.25 M) in acetonitrile for 12 min. 5′-detritylation step was done using 3% trichloroacetic acid in CH_2Cl_2. Oxidation step was done using I_2 (0.02 M) in THF/pyridine/H_2O solution.

For gel electrophoresis, 10X Tris/Borate/EDTA (TBE) buffer was purchased from Fisher Scientific Company L.L.C. (Waltham, MA, USA) and used with proper dilution. 30% Arcylamide/Bis-arcylamide solution (29:1) was purchased from Bio-Rad Laboratories, Inc. (Hercules, CA, USA). GeneRuler 1 kb Plus DNA Ladder (cat.# FERSM1331) was purchased from Fisher Scientific. High resolution ESI-MS spectra of RNA oligonucleotides were acquired by Novatia, LLC.

Molecular weights of the RNA samples were calculated using ChemDraw software. They were experimentally confirmed by Novatia, LLC by Electrospray Ionization Mass Spectrometry (ESI-MS) using Orbitrap Mass spectrometer.

DNA target sequence: 5’-GGTCGAGCTGGACGGCGACG-3’
F-Q probe: 5′-FAM-TTTTTTTTT-BHQ1-3

FAM (6-carboxyfluorescein; excitation/emission: 495/520 nm) and BHQ1 (Black Hole Quencher 1; quenching range: 480–580 nm) were used in the F–Q probe.

UV-melting temperature (T_m) study. 20-nt-RNAs (0.5 μM) along with the complimentary DNA (0.5 μM) were dissolved in a buffer containing 10 mM sodium phosphate (pH = 7.0) and 100 mM

NaCl. The prepared mixtures were first denatured at 95 °C for 3 min, slowly cooled to room temperature, and then kept at 4 °C for 2 h. After that, the samples were degassed 5 min at 400 mmHg vacuum and transferred to quartz cuvettes for absorption recording. The *T*m study was performed on an Agilent Cary UV/Vis spectrophotometer with a temperature controller. The absorptions of each sample at 260 nm were recorded while experiencing 3 heating-cooling cycles from 15 to 95 °C at a rate of 0.5 °C /min. Experiments are performed in triplicate.

CRISPR-Cas12a-crRNA reaction with F-Q probe. All Cas12a–crRNA complexes were prepared at a 1:1.2 molar ratio of Cas12a to crRNA. For each complex, 0.5 µL of Cas12a (in glycerol) was mixed with the appropriate volume of crRNA to achieve final concentrations of 1.0 µM Cas12a and 1.2 µM crRNA in 1X NEB Buffer 2.1. Complex formation was carried out by incubating the mixture at 37 °C for 30 minutes.

Next, 2.4 µL of the prepared Cas12a–crRNA complex (1.0 µM) was mixed with 6 µL of target DNA (100 nM) in 45.6 µL of 1X NEB Buffer 2.1 and incubated at 37 °C for 30 minutes using a Bio-Rad T100™ Thermal Cycler. Following this incubation, 6 µL of 1.0 µM F–Q probe was added, bringing the final reaction volume to 60 µL, with final concentrations of 10 nM target DNA, 40 nM Cas12a–crRNA complex, and 100 nM F–Q probe. For experiments modifying CRISPR-Cas12a-crRNA complex, 1X NEB Buffer 2.1 was used to replace missing volume. Endpoint fluorescence readings (λ_{ex}c = 495 nm, λ_{em} = 520 nm) were recorded every 10 seconds over a 40-minute period. Fluorescence spectra were also collected 40 minutes after addition of the F–Q probe. Data analysis was performed using the allosteric sigmoidal curve fit using GraphPad Prism (GraphPad Software, Boston, MA) software. Initial slope calculations were performed by dividing change in fluorescence intensity over a 5-minute interval by the number of minutes.

DNA target and the F-Q probe were obtained from Integrated DNA Technologies, Inc. (Coralville, IA, USA). An Agilent BioTek Synergy H1 Multimode Plate Reader was used for microplate fluorescence measurements. 96-well half-area microwell plates (catalog #29444-316) were obtained from VWR. EnGen Lba Cas12a (Cpf1) (Cat. #Mo653T), NEBuffer 2.1 10X reaction buffer (Cat. #B6002S), and Monarch PCR & DNA Cleanup Kit (5 µg) (Cat. #T1030S) were sourced from New England BioLabs Inc. (Ipswich, MA, USA).

Note: No unexpected or unusually high safety hazards were encountered during the experiments.

Table S1. The RNA sequences synthesized and used.

RNA	Sequence (5' - 3')	Calculated MW (Da)	Observed MW (Da)
**crRNA1**	5’-AAUUUCUACUCUUGUAGAUCGUCGCCGUCCAGCUCGACC-3’	12294.6	12294.6
**crRNA2**	5’-AAUUUCUACUCUUGUAGAUCGUCGCCGUCCm^6AGCUCG m^6ACC-3’	12328.5	12328.5
**crRNA3**	5’-AAUUUCUACUCUUGUAGAUCGUCGCCGUCCAG m^5CUm^5CGAm^5CC-3’	12342.4	12343.4
**crRNA4**	5’-AAUUUCUACUCUUGUAGAUCGUCGCm^5CGUm^5Cm^5CAGCUCGACC-3’	12342.4	12343.4
**crRNA5**	5’-AAUUUCUACUCUUGUAGAUm^5CGUm^5CGm^5CCGUCCAGCUCGACC-3’	12342.4	12343.3
**20-nt-crRNA1**	5’-CGUCGCCGUCCAGCUCGACC-3’	6289.9	6289.9
**20-nt-crRNA2**	5’-CGUCGCCGUCCm^6AGCUCGm^6ACC-3’	6317.9	6317.9
**20-nt-crRNA3**	5’-CGUCGCCGUCCAGm^5CUm^5CGAm^5CC-3’	6334.9	6335.1
**20-nt-crRNA4**	5’-CGUCGCm^5CGUm^5Cm^5CAGCUCGACC-3’	6334.9	6334.9
**20-nt-crRNA5**	5’-m^5CGUm^5CGm^5CCGUCCAGCUCGACC-3’	6334.9	6334.9

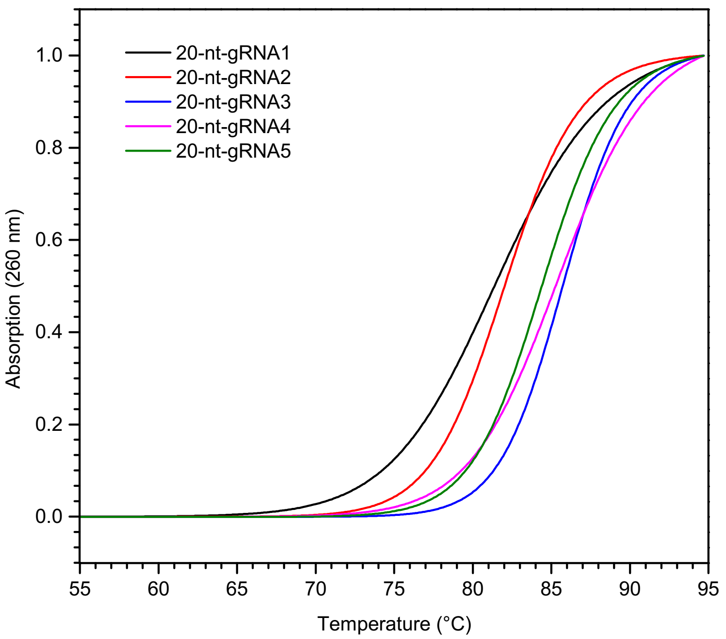

Figure S1. Normalized UV-melting curves of 20-nt guide region of crRNA containing either m^6A or m^5C modifications and the complementary DNA.

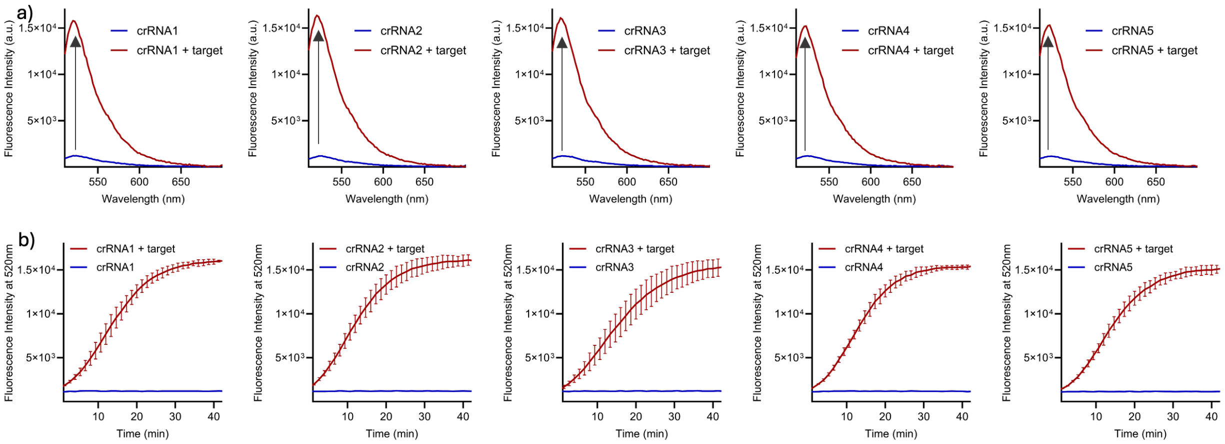

Figure S2. Activation of Cas12a with crRNAs containing m^6A and m^5C modifications. (a) Fluorescence spectra 40 min after addition of the F–Q probe, with and without target DNA. (b) Fluorescence kinetics at 520 nm over 40 min, showing that all crRNAs activate Cas12a, with only slight differences in reaction rates. The experiments were performed in triplicate and data are presented as mean ± SD.

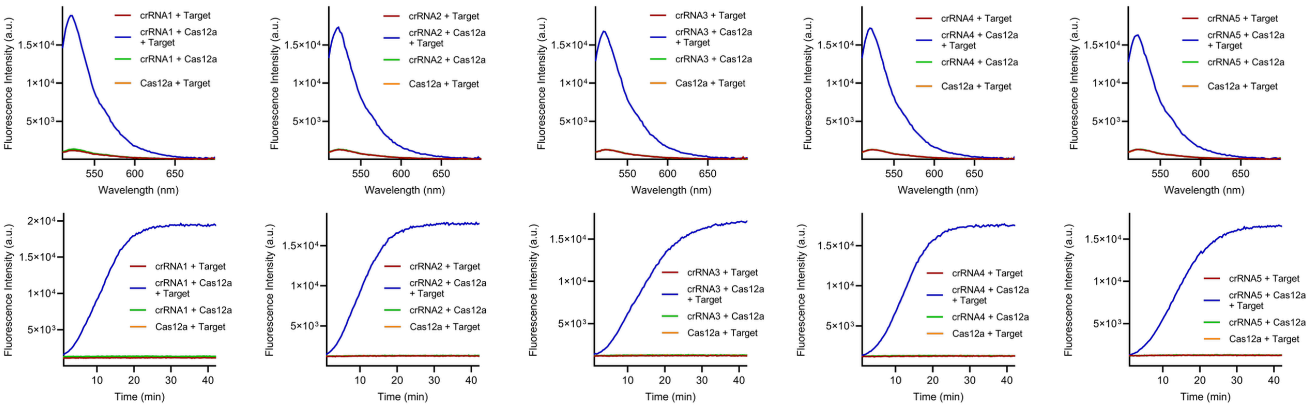

Figure S3. Fluorescence spectra and kinetics of Cas12a nuclease activity with all crRNAs (crRNA1–crRNA5) containing m^{6}A and m^{5}C modifications. Experiments were performed under four conditions: (i) Cas12a with crRNA only (Cas12a–crRNA complex), (ii) Cas12a with target only (no crRNA), (iii) crRNA with target only (no Cas12a), and (iv) Cas12a, crRNA, and target (Cas12a–crRNA–target complex). Fluorescence recovery is observed only when all three components are present.

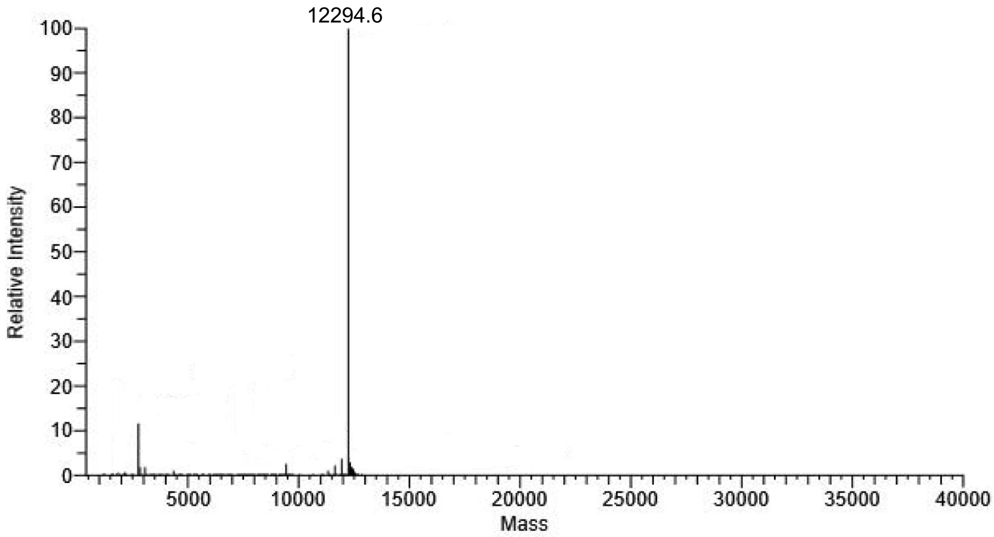

Figure S4. ESI-MS spectrum of **crRNA1**.

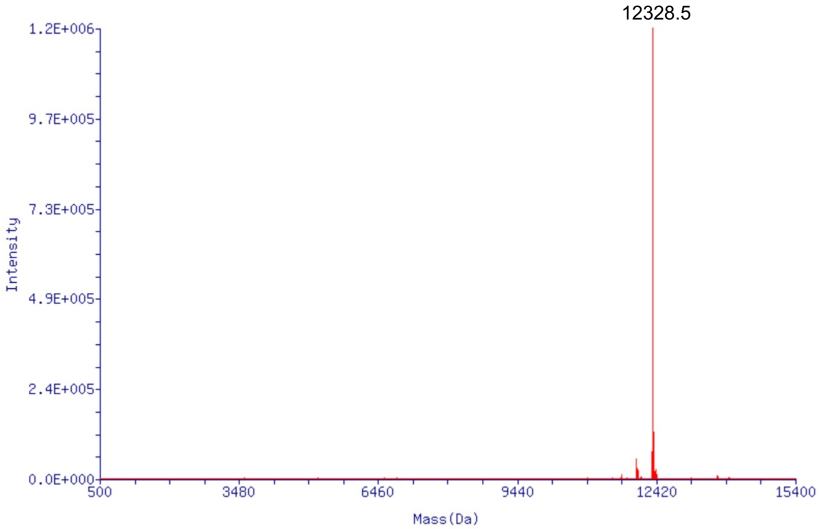

Figure S5. ESI-MS spectrum of **crRNA2**.

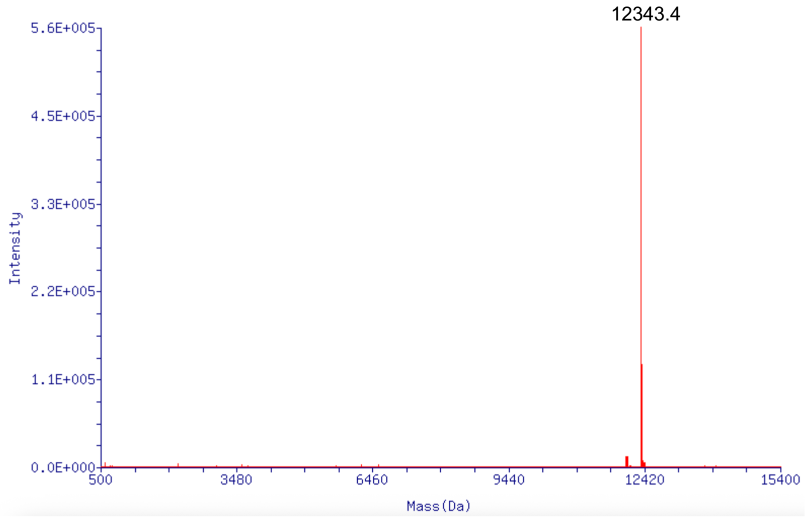

Figure S6. ESI-MS spectrum of **crRNA3**.

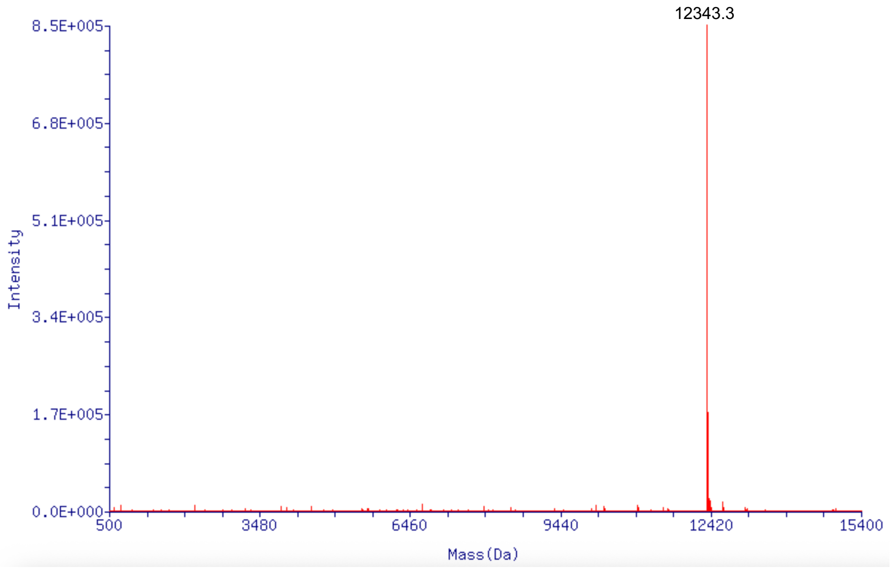

Figure S7. ESI-MS spectrum of **crRNA4**.

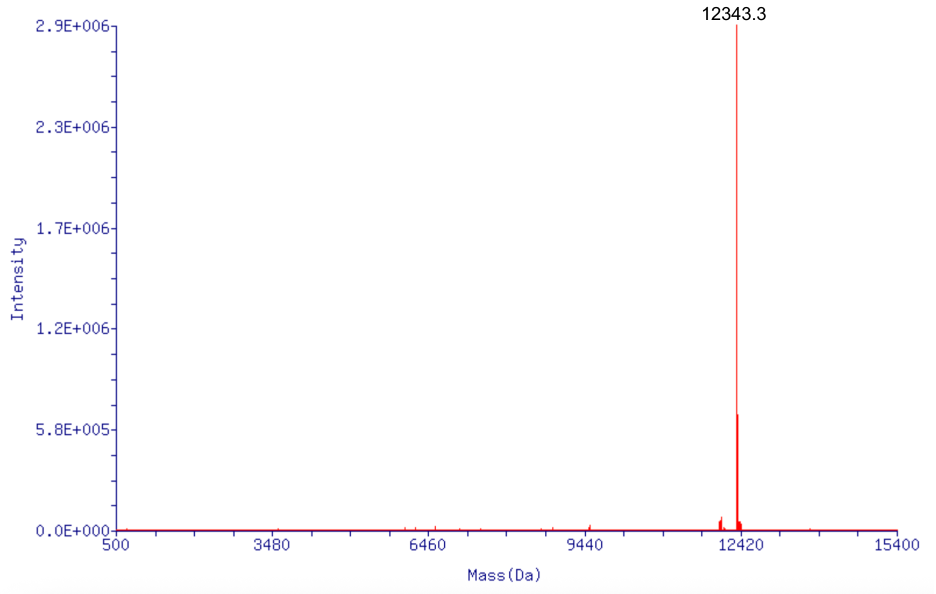

Figure S8. ESI-MS spectrum of **crRNA5**.

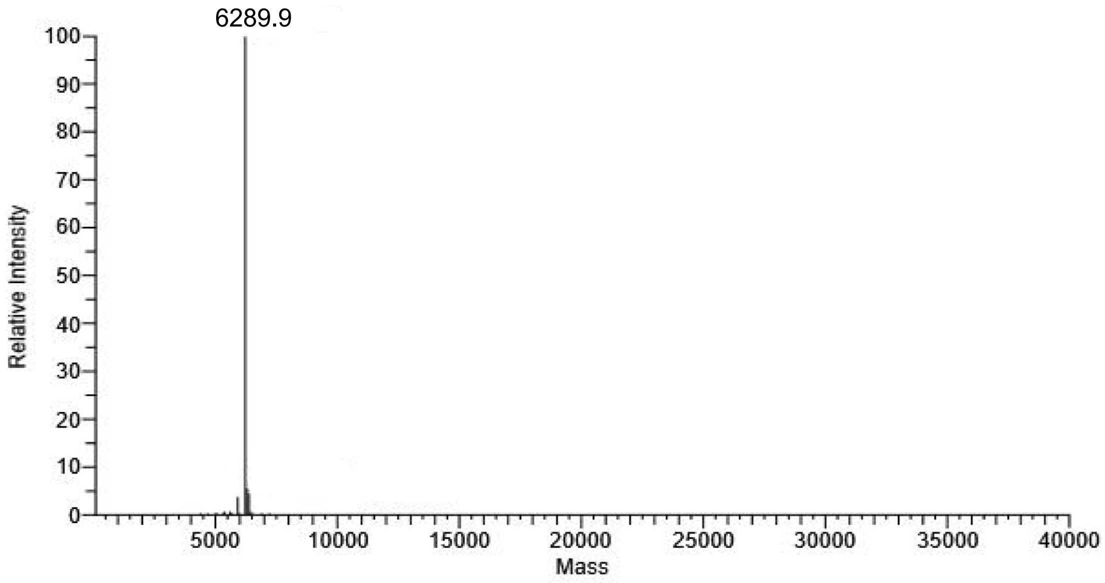

Figure S9. ESI-MS spectrum of **20-nt-crRNA1**

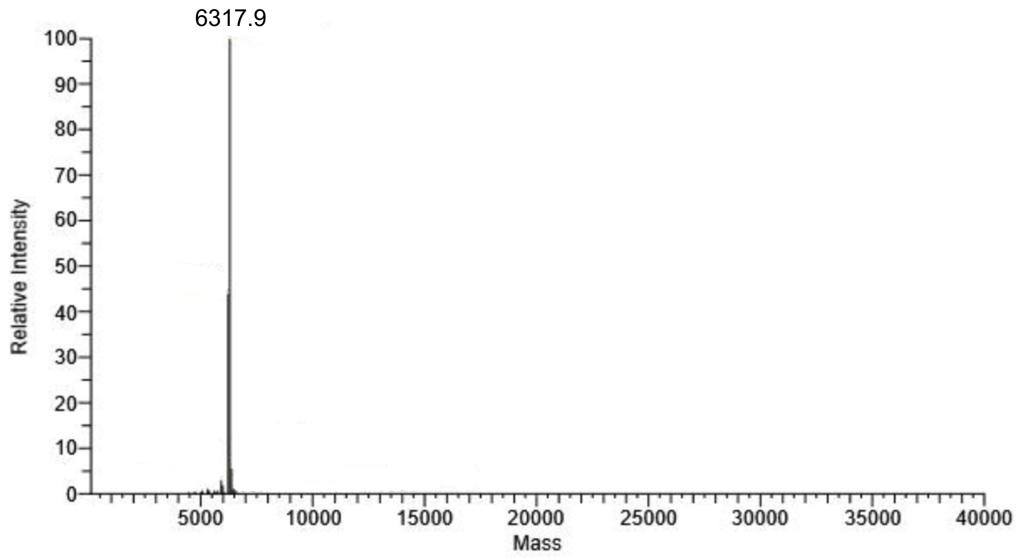

Figure S10. ESI-MS spectrum of **20-nt-crRNA2**

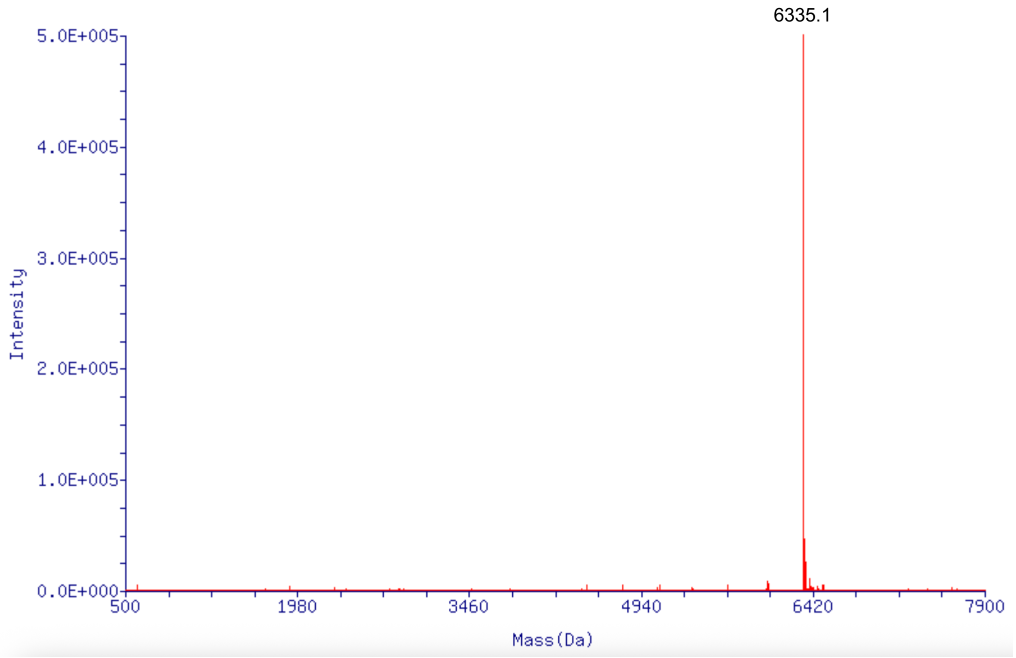

Figure S11. ESI-MS spectrum of **20-nt-crRNA3**

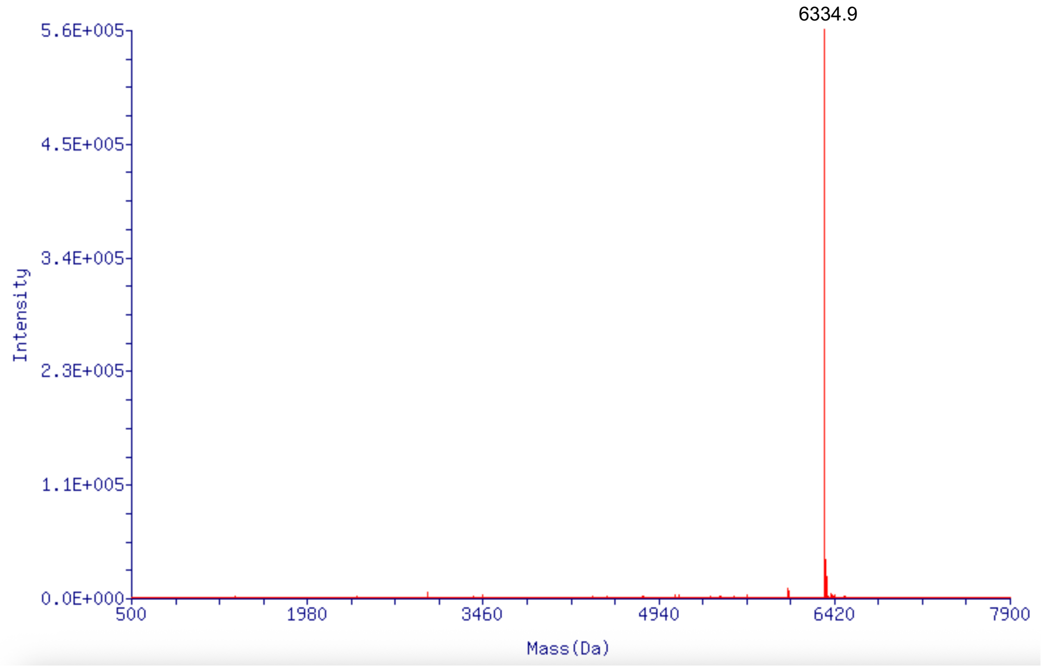

Figure S12. ESI-MS spectrum of **20-nt-crRNA4**

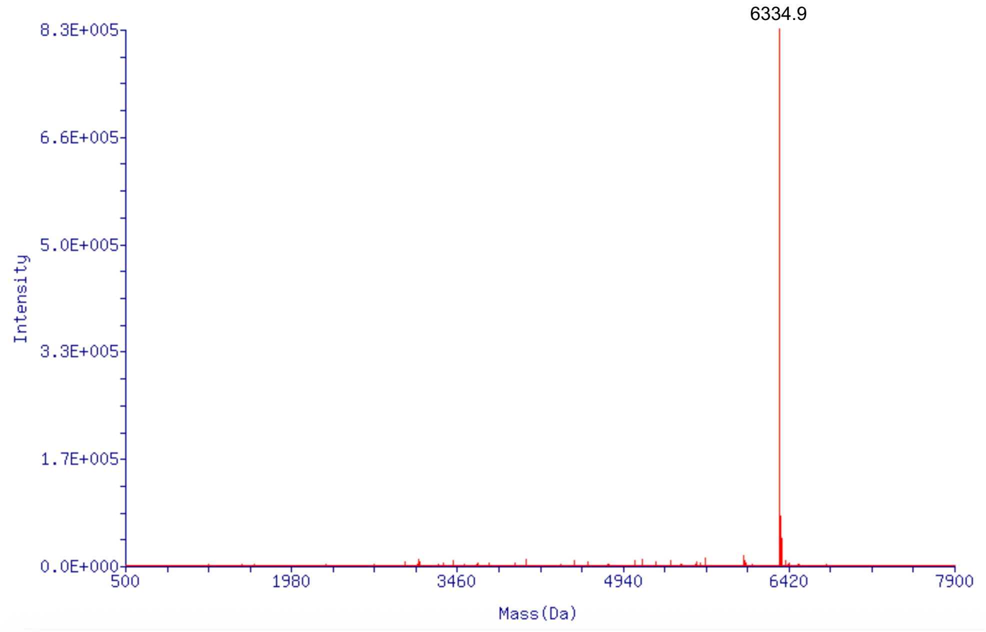

Figure S13. ESI-MS spectrum of **20-nt-crRNA5**

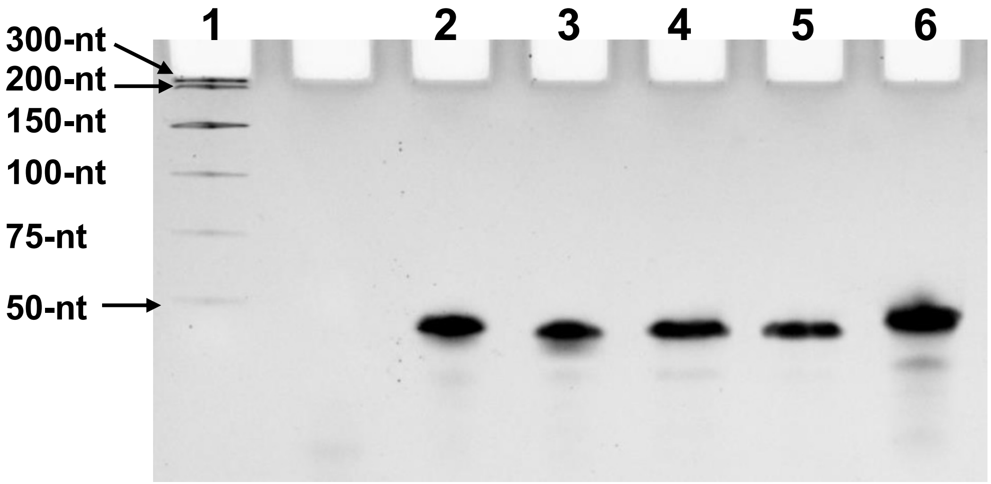

Figure S14. PAGE analysis of purified crRNAs. *Lane 1:* RNA ladder; *Lane 2:* **crRNA1**; *Lane 3:* **crRNA2**; *Lane 4:* **crRNA3**; *Lane 5:* **crRNA4**; *Lane 6:* **crRNA5**.
